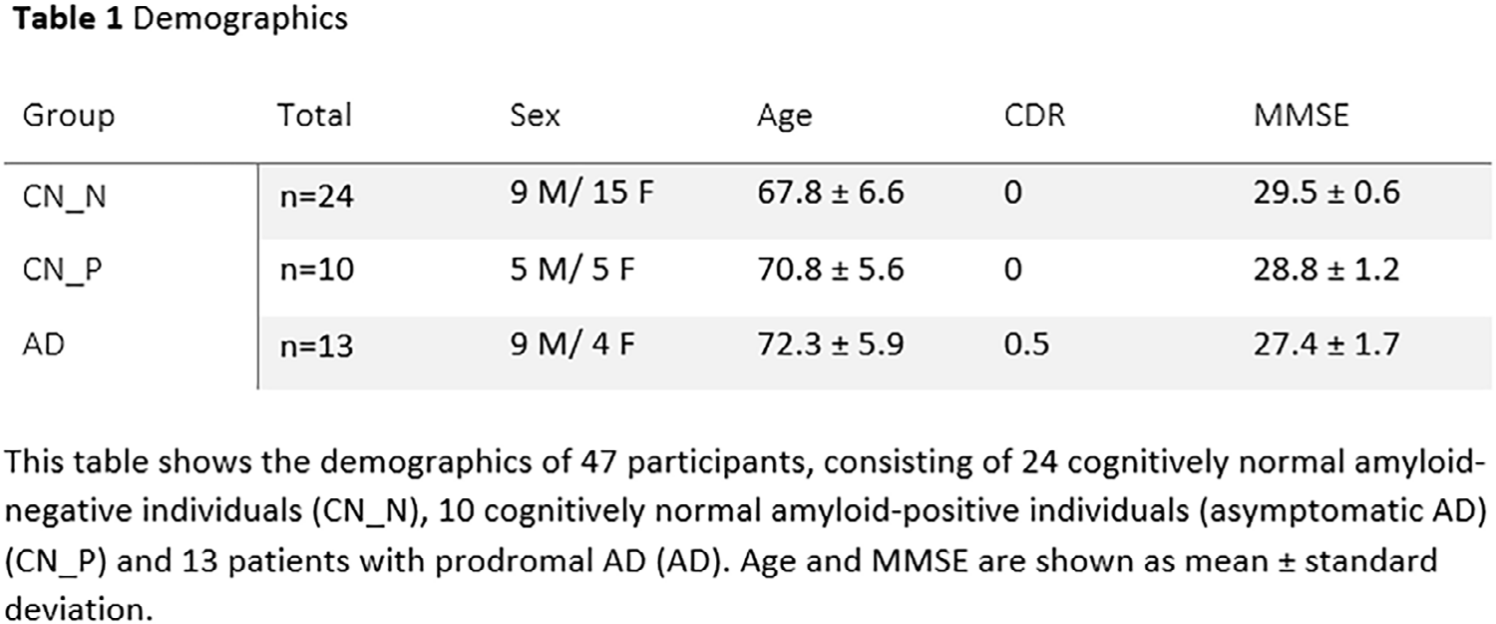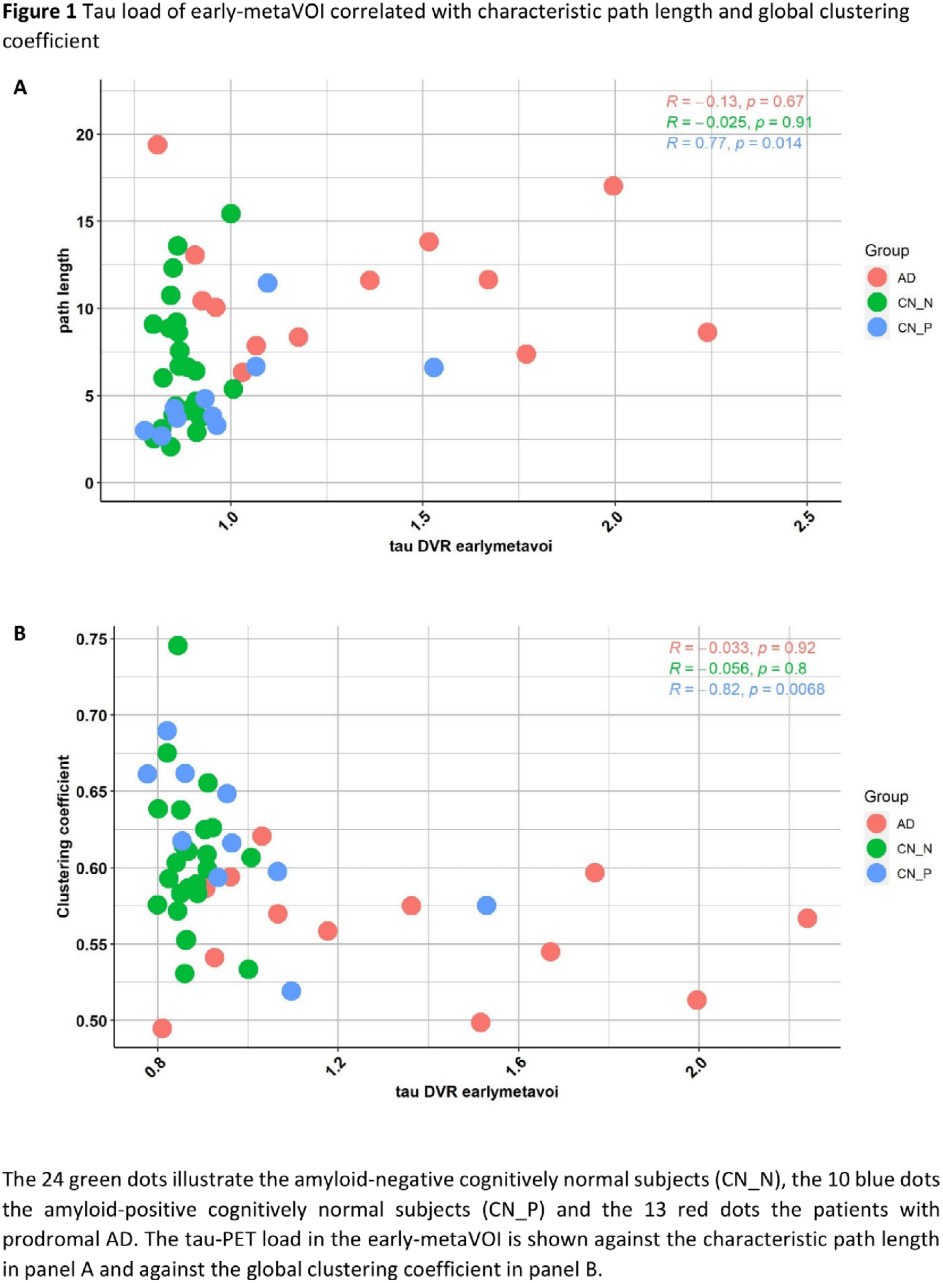# Effect of tau on global graph measures from high‐density EEG in preclinical and prodromal stage of Alzheimer disease

**DOI:** 10.1002/alz.091326

**Published:** 2025-01-09

**Authors:** Laure Spruyt, Tjaša Mlinarič, Mariska Reinartz, Marc Van Hulle, Koen Van Laere, Patrick Dupont, Rik Vandenberghe

**Affiliations:** ^1^ Laboratory for Cognitive Neurology, KU Leuven, Leuven Belgium; ^2^ Alzheimer Research Centre KU Leuven, Leuven Brain Institute, Leuven Belgium; ^3^ Laboratory for Neuro‐ and Psychophysiology, KU Leuven, Leuven Belgium; ^4^ KU Leuven, Leuven Belgium; ^5^ University Hospitals Leuven, Leuven Belgium

## Abstract

**Background:**

In Alzheimer disease (AD) changes in the small world network have been described, which can be considered as the balance between local connectivity, exhibited with the “clustering coefficient”, and global integration, investigated by “characteristic path length”. With high‐density EEG, we examined the effect of early spread of tau aggregates on these global graph measures.

**Method:**

This study includes 47 participants who underwent a 100‐minute dynamic ^18^F‐MK6240 PET‐scan and a five‐minutes eyes closed 128‐channel resting‐state EEG. The total group consisted of 24 cognitively normal amyloid‐negative individuals (CN_N), 10 cognitively normal amyloid‐positive individuals (asymptomatic AD) (CN_P) and 13 patients with prodromal AD (Table 1). We calculated in the alpha frequency band the average undirected weighted Phase‐Lag‐Index and used this as weights for the graph and analyzed the global graph measures characteristic path length and clustering coefficient in sensor‐space. For both the whole group as for the constituent subgroups, we examined the Spearman correlation between these global graph measures and tau‐PET load in an a priori defined early‐metaVOI, comprised of the entorhinal and perirhinal cortex, hippocampus, parahippocampus and fusiform cortex. To investigate the difference between tau‐PET load, clustering coefficient and path length in the 3 subgroups, we applied a Kruskal‐Wallis test.

**Result:**

There was a significant correlation of tau‐PET load with the characteristic path length (r=0.33; p=0.03) and also with the global clustering coefficient (r=‐0.39; p=0.007) in the whole group analysis. When considering the asymptomatic AD group separately, there was also a significant correlation of tau‐PET load with the characteristic path length (r=0.77; p=0.01) and with the global clustering coefficient (r=‐0.82; p=0.007). There was no significant correlation within the CN_N subgroup (path length r=‐0.03; p=0.91; clustering coefficient (r=‐0.06; p=0.80)), neither in the prodromal AD subgroup (path length r=‐0.13; p=0.67; clustering coefficient (r=‐0.03; p=0.92)) (Figure 1). There was a significant difference in tau‐PET load between the subgroups CN_N and prodromal AD (p<0.025) and in clustering coefficient and path length between the CN_N and the CN_P group versus the prodromal AD subgroup (p<0.025).

**Conclusion:**

In the asymptomatic stage of AD, focal tau‐PET load in medial temporal cortex is associated with global electrophysiological measures of network disintegration.